# Publisher Correction: Aerodynamic generation of electric fields in turbulence laden with charged inertial particles

**DOI:** 10.1038/s41467-018-04545-6

**Published:** 2018-05-21

**Authors:** M. Di Renzo, J. Urzay

**Affiliations:** 1 Center for Turbulence Research, Stanford University, Stanford, CA 94305 USA; 2 Dipartimento di Meccanica, Matematica e Management, Politecnico di Bari, Bari, 70125 Italy

Correction to: *Nature Communications* 10.1038/s41467-018-03958-7; published online 26 April 2018

The original version of this Article contained an error in the last sentence of the second paragraph of the ‘Atmospheric rarefaction effects’ section of the Results, which incorrectly read ‘The other one emulates the rarefied, CO_2_-rich Martian atmosphere (*μ*_♂_ = 1.3 × 10^−5^ N s m^−2^) at 6.9 mbar and 210 K, which gives *ρ*_♂_ = 1.6 × 10^−12^ kg m^−3^.’ The correct version states ‘*ρ*_♂_ = 1.6 × 10^−2^ kg m^−3^’ in place of ‘*ρ*_♂_ = 1.6 × 10^−12^ kg m^−3^’. This has been corrected in both the PDF and HTML versions of the Article.

